# Olfactory Bulb Glomerular NMDA Receptors Mediate Olfactory Nerve Potentiation and Odor Preference Learning in the Neonate Rat

**DOI:** 10.1371/journal.pone.0035024

**Published:** 2012-04-04

**Authors:** Rebecca Lethbridge, Qinlong Hou, Carolyn W. Harley, Qi Yuan

**Affiliations:** 1 Biomedical Sciences, Faculty of Medicine, Memorial University of Newfoundland, St. John’s, Canada; 2 Department of Psychology, Faculty of Science, Memorial University of Newfoundland, St. John’s, Canada; Centre national de la recherche scientifique, France

## Abstract

Rat pup odor preference learning follows pairing of bulbar beta-adrenoceptor activation with olfactory input. We hypothesize that NMDA receptor (NMDAR)-mediated olfactory input to mitral cells is enhanced during training, such that increased calcium facilitates and shapes the critical cAMP pattern. Here, we demonstrate, *in vitro*, that olfactory nerve stimulation, at sniffing frequencies, paired with beta-adrenoceptor activation, potentiates olfactory nerve-evoked mitral cell firing. This potentiation is blocked by a NMDAR antagonist and by increased inhibition. Glomerular dishinhibtion also induces NMDAR-sensitive potentiation. *In vivo*, in parallel, behavioral learning is prevented by glomerular infusion of an NMDAR antagonist or a GABA_A_ receptor agonist. A glomerular GABA_A_ receptor antagonist paired with odor can induce NMDAR-dependent learning. The NMDA GluN1 subunit is phosphorylated in odor-specific glomeruli within 5 min of training suggesting early activation, and enhanced calcium entry, during acquisition. The GluN1 subunit is down-regulated 3 h after learning; and at 24 h post-training the GluN2B subunit is down-regulated. These events may assist memory stability. *Ex vivo* experiments using bulbs from trained rat pups reveal an increase in the AMPA/NMDA EPSC ratio post-training, consistent with an increase in AMPA receptor insertion and/or the decrease in NMDAR subunits. These results support a model of a cAMP/NMDA interaction in generating rat pup odor preference learning.

## Introduction

Odor preference learning in the neonate rat is a robust cAMP/PKA/pCREB-dependent mammalian appetitive learning model [1]–[4] in which the mechanisms for learning have been localized to the olfactory bulb [2], [5]–[10]. Rat pups are dependent on proximity to the dam for survival in the first week and use odor, as do human neonates, to guide maternally-reinforced approach behavior [11]. In rodent experiments, an odor (e.g. peppermint) is paired with reward to induce an odor preference [12], [13]. An odor preference is readily induced when odor is paired with natural reinforcing stimuli such as repeated gentle stroking [12], [13] or intraoral milk infusion [14], [15]. At a more mechanistic level, odor preference learning can also be produced by pairing odor with injections of the beta-agonist isoproterenol [7]. Natural reinforcing stimuli and isoproterenol interact additively [16]. Importantly for the present investigation, activation of β-adrenoceptors solely in the olfactory bulb paired with odor presentation is necessary and sufficient for odor preference learning [7]. The circuitry for this intrabulbar learning model is relatively simple. The olfactory nerve, carrying odor information, contacts mitral cell (MC) dendrites in glomeruli at the outer edge of the olfactory bulb. MCs (together with deep tufted cells) are the transducers for odor information to the brain. They receive odor input as a function of the strength of glomerular connections, their responses are shaped and modulated by local inhibitory interneurons, and their axonal output constitutes the bulbar odor representation projected through the lateral olfactory tract to the cortical area.

Our model of the cellular substrates of odor preference learning assigns an important role to N-methyl-D-aspartate receptors (NMDARs) as mediators of the pairing between odor and reward in MCs [4]. Calcium entering MCs via NMDAR activation is hypothesized to interact with calcium-sensitive adenylate cyclase in MCs to critically shape the intracellular cAMP signal as first suggested by Yovell and Abrams [17], and shown in the work of Cui et al [1]. cAMP-mediated phosphorylation of MC NMDARs may provide a positive feedback loop for these effects. The role of NMDARs in odor preference learning has, however, not been well understood.

Previous work established that pairing the β-adrenoceptor activator, isoproterenol, with olfactory nerve (ON) stimulation in anesthetized rat pups produces an enduring enhancement of the ON-evoked glomerular field potential [18]. Odor preference training also produces an increase in MC pCREB activation [2]. Increasing MC pCREB levels using viral CREB lowers the learning threshold and attenuating MC pCREB increases prevents learning [3]. Recently, in an *in vitro* model of odor learning, it was shown that theta burst stimulation (TBS) of the ON, approximating sniffing frequency, paired with β-adrenergic receptor activation using isoproterenol produces increased MC calcium signaling [19], consistent with our model. The present experiments, first test the role of NMDARs in this novel *in vitro* model, and then explore their role *in vivo* in early odor preference learning.

In the *in vivo* experiments, PKA modulation of the GluN1 subunit was imaged following training and new intrabulbar experiments, using MC pCREB activation to index selective peppermint odor MC recruitment, were carried out to establish cannulae placements for localized glomerular infusion of the NMDAR antagonist, D-APV. Behavioral experiments with localized infusions assessed the hypotheses that glomerular NMDARs and glomerular GABA_A_ receptors are modulated by isoproterenol to induce odor preference learning. Since down-regulation of NMDAR subunits has been reported in *in vitro* plasticity models [20] and during development [21], the down-regulation of olfactory bulb NMDAR subunits with odor preference learning was probed. Finally, *ex vivo* experiments, directly measuring AMPA/NMDA currents in MCs from trained rat pups, assessed the cellular locus of learning. Taken together the results strongly support a role for glomerular NMDA receptors in the acquisition of odor preference learning and suggest a subsequent downregulation of NMDA-mediated plasticity following learning.

## Results

### MC Spike Potentiation by Pairing Isoproterenol and TBS is NMDAR-dependent

**Figure 1:**
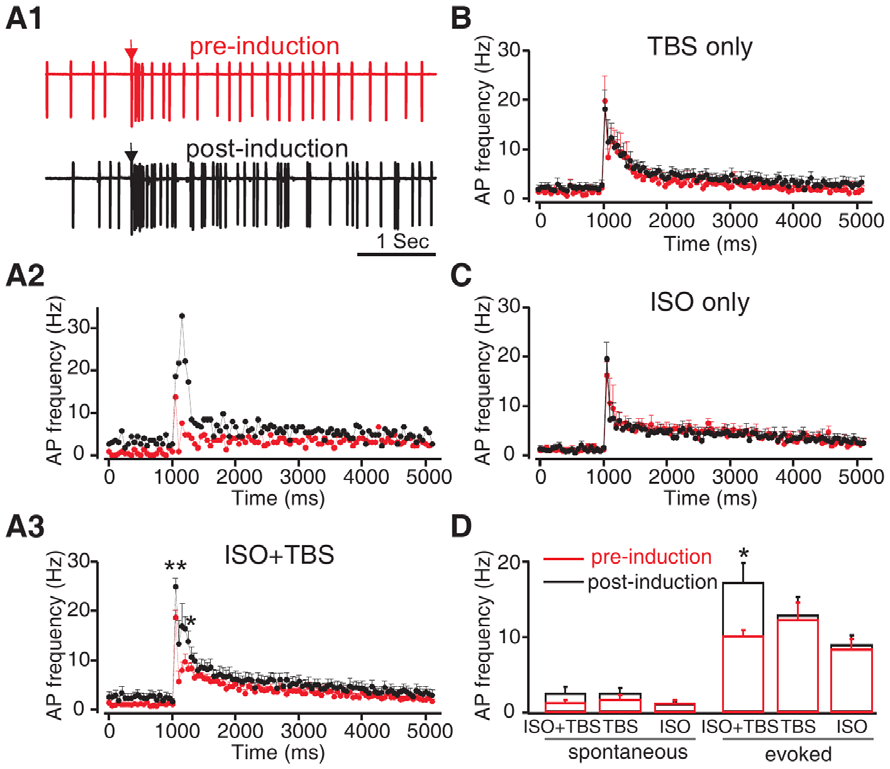
Isoproterenol (ISO) pairing with olfactory nerve theta burst stimulation (TBS) induced mitral cell (MC) spike potentiation. ***A1*** **.** Single MC spiking patterns before and 30 min following TBS induction in the presence of the β-adrenoceptor agonist isoproterenol (10 µM). Arrow indicates the time of the single olfactory nerve test stimulus. ***A2&A3*** **.** Peristimulus spike frequency histograms (binning 50 ms) under the control condition (red) and 20–30 min after TBS induction of the example cell in *A1* (*A2*); and of the average of *n* = 10 cells in the same condition (*A3*). ***B*** **.** Peristimulus spike frequency histograms under the control condition and 20–30 min after TBS induction in the absence of ISO (*n* = 10). ***C*** **.** Peristimulus spike frequency histogram under the control condition and 20–30 min following a brief (5 min) application of ISO. ***D*** **.** Histogram comparing mean spike frequencies during the 250 ms intervals before (spontaneous) and after the olfactory nerve stimulation (evoked) under control conditions (red) and 20–30 min post-inductions (black). **p<0.01 *p<0.05. Error bars, mean±SEM.

Previous research supports an enhanced MC excitation model for early odor preference learning [4], [19]. Our recent report [19] established an *in vitro* slice preparation that mimics the *in vivo* learning conditions. Using acute olfactory bulb slices from young rats, odor input was mimicked *in vitro* by TBS of the ON, and the modulation of MC responses to TBS alone and in conjunction with bath application of the β-adrenoceptor agonist, isoproterenol, was assessed. Previously, pairing 10 µM isoproterenol with TBS led to a potentiation of MC somatic calcium transients, which was not seen with TBS alone, or isoproterenol alone [19], although TBS alone produced long-term potentiation (LTP) of the glomerular field EPSP. Somatic calcium transients reflect spikes in various principle neurons including MCs [22]–[25] and are of particular interest as they suggested increased MC throughput. Since the evoked calcium response was normalized to the baseline level, the result implied two scenarios: first, only the TBS+ISO induction enhanced MC evoked responses; second, the TBS+ISO induction enhanced the ratio of evoked/spontaneous responses. Here we directly measure MC spikes using loose-patch recording of MCs and show that throughput is increased as indexed by evoked spiking. Figure 1 shows that bath application of isoproterenol during TBS (isoproterenol was washed in 5–10 min before the TBS induction and washed out immediately after), induced MC spike potentiation to ON input consistent with the previous report [19]. As shown in Figure 1*A*, MCs show spontaneous spiking at an average frequency of less than 5 Hz (Figure 1*A* *1–3*). As a side note, we observed some cells with little spontaneous spiking that appeared healthy under DIC and responded well to ON stimulation. MCs recorded *in vivo* can also show a lack of spontaneous spiking using similar extracellular recording methods [26]. Stimulation of the ON produces a long evoked response of increased MC spiking activity, which can last for seconds, but the increased spiking is most obvious in the first 250 ms (Figure 1*A* *1–3*). We measured and compared the spontaneous (250 ms prior to stimulation) and evoked (250 ms following stimulation) activities of MCs in the presence of isoproterenol before TBS induction and those 20–30 min following TBS induction. Evoked (mean baseline  =  10.15±0.79 Hz), but not spontaneous (mean baseline =  1.24±0.37 Hz) MC spiking, was significantly increased 20–30 min following pairing of TBS and isoproterenol (*n* = 10; Figure 1*A* *3,D*). There was an average 69% increase in evoked spiking. Among these cells, eight out of the ten cells showed increased evoked spiking. These include two cells that showed decreased, and one cell that showed unchanged, spontaneous spiking. When the 250 ms interval was subdivided into a 0–50 ms AMPA component and a 50–250 ms NMDA component [23], both components were significantly increased (AMPA: from 18.73±1.49 to 25±1.68, *t* = 3.633, *p* = 0.003; NMDA: from 8±0.85 to 15.2±2.99; *t* = 2.359, *p* = 0.04). The increase in MC spiking is specific to the pairing of TBS and isoproterenol since TBS alone (*n* = 10; *t* = 0.264, *p* = 0.798; Figure 1*B,D*) or isoproterenol alone (*n* = 10; *t* = 0.954, *p* = 0.377; Figure 1*C,D*) failed to enhance MC evoked spikes.

**Figure 2:**
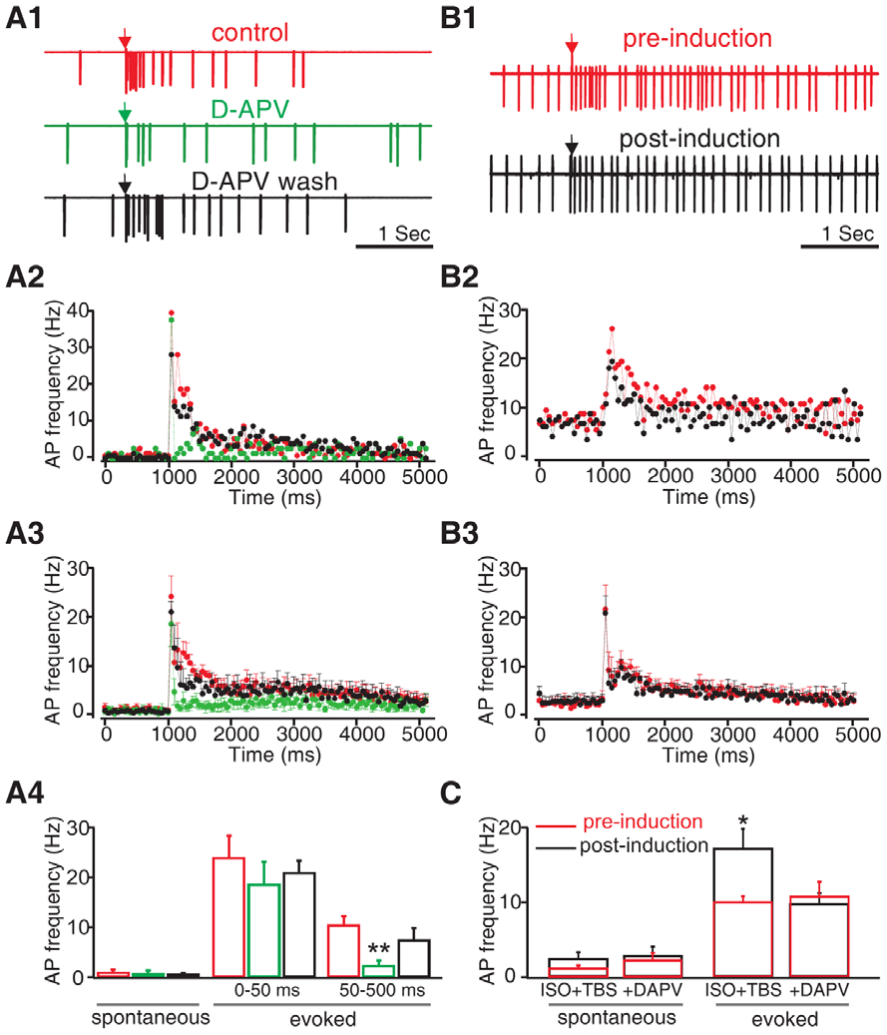
NMDA receptor antagonist D-APV blocked ISO+TBS induced MC spike potentiation. *A1-A4*. D-APV effect on MC spiking. ***A1*** **.** Single MC spiking patterns before, in the presence of, and 30 min following D-APV (50 µM) bath application. Arrow indicates the time of the single olfactory nerve test stimulus. ***A2&A3*** **.** Peristimulus spike frequency histograms (binning 50 ms) under the control condition (red), in the presence of D-APV (green) and 20–30 min following D-APV washout of the example cell in *A1* (*A2*); and of the average of *n* = 4 cells in the same condition (*A3*). ***A4*** **.** Histogram comparing mean spike frequencies during the 250 ms intervals before (spontaneous) and at two time intervals after the olfactory nerve stimulation (evoked) under control conditions (red), during D-APV application (green) and 20–30 min following D-APV washout (black). ***B1-B3*** **.** D-APV bath application blocked MC spike potentiation induced by paring ISO with TBS. ***B1*** **.** Single MC spiking patterns before and 30 min following TBS induction in the presence of ISO and D-APV. ***B2&B3*** **.** Peristimulus spike frequency histograms under the control condition and 20–30 min after TBS induction in the presence of ISO and D-APV of the example cell in *B1* (*B2*); and of the average of *n* = 6 cells (*B3*). **C.** Histogram comparing mean spike frequencies during the 250 ms intervals before (spontaneous) and after the olfactory nerve stimulation (evoked) under control conditions (red) and 20–30 min post-induction (black). **p<0.01. Error bars, mean±SEM.

This result suggests that the pairing of isoproterenol with TBS specifically enhances MC excitation to ON input. We next explored whether the pairing of isoproterenol with TBS-induced MC potentiation is NMDAR-dependent. We tested whether D-APV application has an effect on the MC spiking potentiation induced by pairing TBS with isoproterenol. We first examined the acute effect of D-APV itself on the MC spiking pattern. As expected, D-APV bath application did not change the early 0–50 ms component (*n* = 6 for control and D-APV groups, *n* = 4 for D-APV wash; *F*_2,13_ = 0.490, *p* = 0.624). This early component is presumably mediated by AMPA receptors [27] as NBQX (40 µM), an AMPA receptor antagonist, fully abolished this component in the presence of D-APV (*n* = 3, data not shown). Furthermore, D-APV application did not change the spontaneous firing rate of MCs (1.06±0.4 before D-APV and 0.76±0.56 in the presence of D-APV, *n* = 6, *t* = 0.85, *p* = 0.43). However, D-APV dramatically reduced MC-evoked late spikes (50–500 ms) and the effect was reversed after D-APV washout (control: 10.38±1.79Hz; in the presence of D-APV: 2.41±0.92Hz; D-APV wash: 7.5±2.29 Hz; *F*_2,13_ = 6.87, *p* = 0.009; Figure 2*A* *1–4*), suggesting that NMDARs mediate a late component of the synaptic potentials that lead to MC spikes. Addition of D-APV to the bath at the same period with isoproterenol abolished the increase in MC evoked spikes associated with isoproterenol+TBS (pre-induction: 10.87±2.04 Hz; post-induction: 9.75±1.42 Hz; *n* = 6; *t* = 0.820, *p* = 0.450; Figure 2*B* *1–3,C*). D-APV prevented the potentations of both the AMPA and NMDA components.

### β-adrenoceptors May Act Through Disinhibition of MCs from Glomerular Interneurons to Induce NMDAR-dependent Plasticity of MCs

D-APV blocking of MC spike potentiation in this *in vitro* model suggests β-adrenoceptor activation paired with odor input triggers a NMDAR-dependent potentiation of odor-encoding MCs. One route for the β-adrenoceptor-mediated activation of NMDARs on MCs to promote the LTP of MC responses could be disinhibition. The previous *in vitro* study [19] suggested that β-adrenoceptor activation by isoproterenol suppressed evoked EPSCs in periglomerular cells in the olfactory bulb slice. If disinhibition is important in the opening of NMDARs on mitral cells, we expected increasing glomerular inhibition would counteract the isoproterenol effect in potentiating MCs when paired with TBS, and local glomerular disinhibition could, by itself, lead to NMDAR-dependent potentiation of MC spikes.

**Figure 3:**
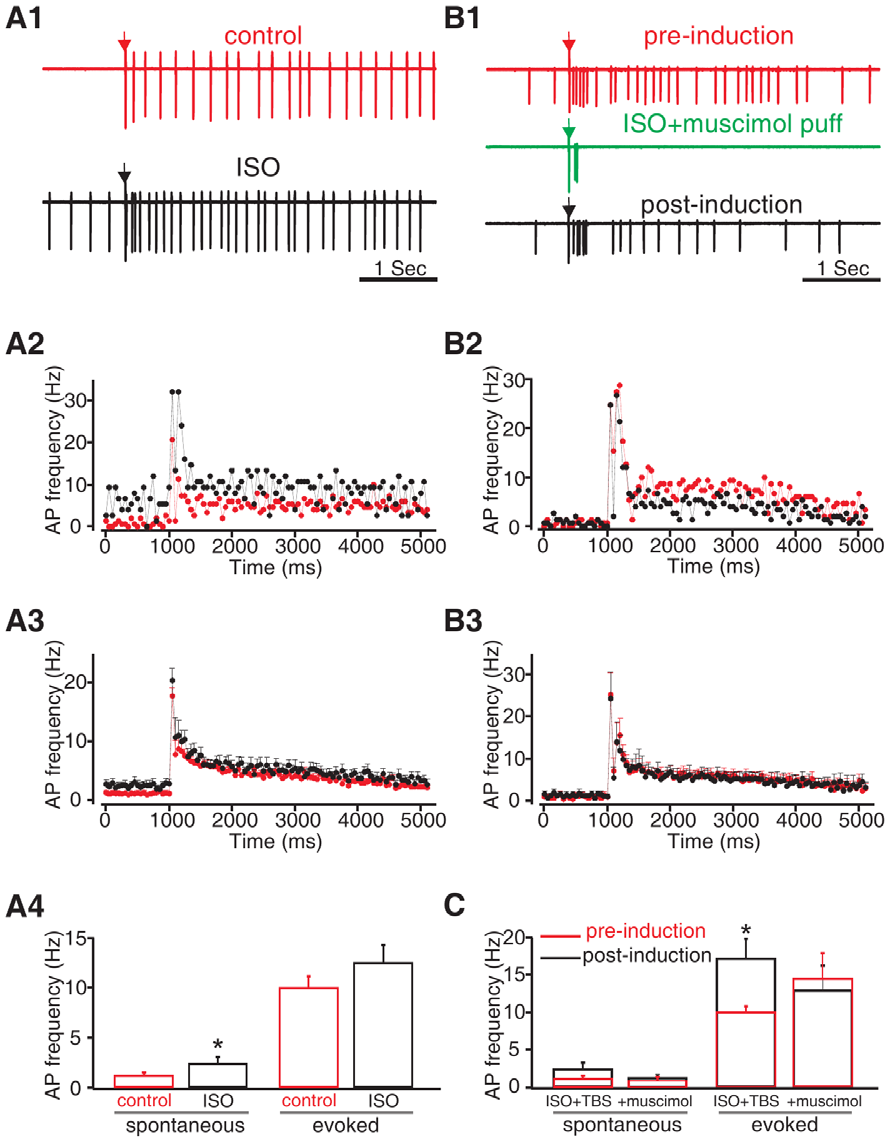
Glomerular application of muscimol blocked ISO+TBS induced MC spike potentiation. *A1-A4*. Acute effect of isoproterenol on MC spiking. ***A1*** **.** Single MC spiking patterns in the control condition and in the presence of ISO bath application. Arrow indicates the time of the single olfactory nerve test stimulus. ***A2&A3*** **.** Peristimulus spike frequency histograms (binning 50 ms) under the control condition (red), in the presence of ISO (black) of the example cell in *A1* (*A2*); and of the average of *n* = 7 cells in the same condition (*A3*). ***A4*** **.** Histogram comparing mean spike frequencies during the 250 ms intervals before (spontaneous) and after the olfactory nerve stimulation (evoked) under control conditions (red) and during ISO application (black). ***B1-B3*** **.** Local puff of the GABAA receptor agonist muscimol (0.2–10 µM) to the glomerular layer adjacent to the stimulation pipette during TBS blocked ISO+TBS induced MC spike potentiation. ***B1*** **.** Single MC spiking patterns before, and 30 min following, TBS induction in the presence of ISO and local muscimol application. ***B2&B3*** **.** Peristimulus spike frequency histograms under the control condition and 20–30 min after TBS induction in the presence of ISO and muscimol of the example cell in *B1* (*B2*); and of the average of *n* = 6 cells (*B3*). **C.** Histogram comparing mean spike frequencies during the 250 ms intervals before (spontaneous) and after olfactory nerve stimulation (evoked) under control conditions (red) and 20–30 min post-inductions (black). *p<0.05. Error bars, mean±SEM.

We first analyzed the acute effect of isoproterenol on MC spiking. A previous study by the Ennis group reported that 10 µM isoproterenol or 10 µM norepinephrine, can cause a reversible inward current (∼50 pA) in MC membrane potentials [28]. Consistent with this inward current effect, our data showed an acute 5 min application of 10 µM isoproterenol resulted in a modest but significant increase in MC spontaneous spikes (pooling the 17 cells exposed to ISO for 5 min in Fig. 1; control: 1.29±0.27 Hz; in the presence of isoproterenol: 2.41±0.64 Hz; *t* = 2.034, *p* = 0.029, Figure 3*A* *1–4*). Twenty to thirty minutes after washout there was no difference in spontaneous activity compared to control baseline however (*n* = 7; *t* = 0.323, *p* = 0.757; see Figure 1*C,D*); arguing against a sustained inward current effect. ON-evoked activity was not altered by acute isoproterenol (control: 17.69±1.39 Hz; in the presence of isoproterenol: 20.31±2.00 Hz; *t* = 1.639, *p* = 0.121, Figure 3*A* *1–4*). We then found that local application of the GABA_A_ receptor agonist muscimol to the glomerular layer surrounding the stimulation pipette counteracted the long-lasting potentiation of MC spikes induced by TBS and isoproterenol pairing. When various concentrations of muscimol (0.2–10 µM) were locally puffed to the glomerular layer, they significantly reduced MC spiking (Figure 3*B* *1*), in most cases abolishing spiking, suggesting glomerular inhibition powerfully “gates” MC activation, possibly through feed-forward inhibitory interneurons [19], [29]. Local puffs of muscimol to the glomerular layer also abolished potentiation of MC evoked spikes induced by TBS and isoproterenol pairing (pre-induction: 14.44±3.52 Hz; post-induction: 13.02±3.24 Hz; *n* = 6; *t* = 0.909, *p* = 0.405; Figure 3*B* *1–3,C*).

**Figure 4:**
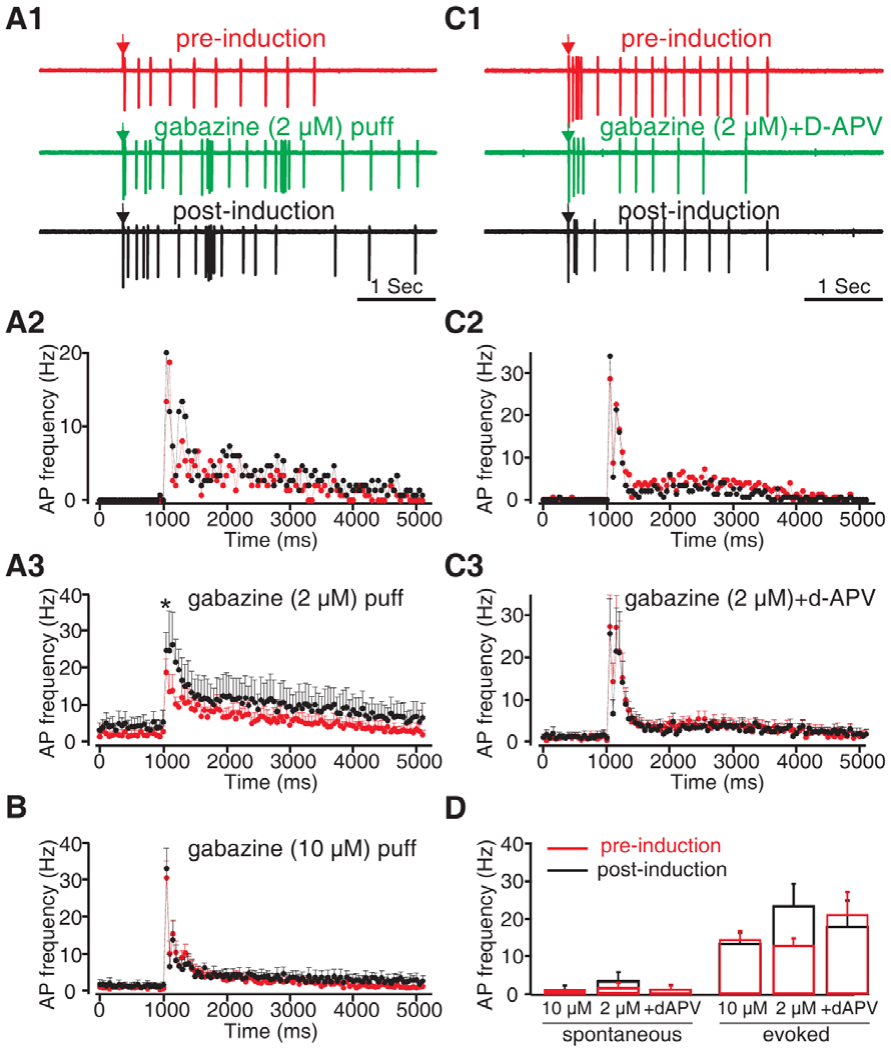
Glomerular disinhibition induced MC spike potentiation and was dose- and D-APV dependent. *A1-A3*. 2 µM gabazine (GABAA antagonist) induced MC spike potentiation following TBS. ***A1*** **.** Single MC spiking patterns in the control condition and in the presence of 2 µM gabazine locally puffed to the glomerular layer. Arrow indicates the time of the single olfactory nerve test stimulus. ***A2&A3*** **.** Peristimulus spike frequency histograms (binning 50 ms) under the control condition (red) and in the presence of 2 µM gabazine (black) of the example cell in *A1* (*A2*), and of the average of *n* = 6 cells in the same condition (*A3*). ***B*** **.** Peristimulus spike frequency histograms showing that a higher dose (10 µM) of gabazine local application failed to induce MC spike potentiation. ***C1-C3*** **.** D-APV application blocked MC spike potentiation induced by 2 µM gabazine. ***C1*** **.** Single MC spiking patterns before and 30 min following TBS induction in the presence of a 2 µM gabazine and 500 µM D-APV local puff. ***C2&C3.*** Peristimulus spike frequency histograms under the control condition and 20–30 min after TBS induction in the presence of gabazine and D-APV of the example cell in *C1* (*C2*); and of the average of *n* = 7 cells (*C3*). **D.** Histogram comparing mean spike frequencies during the 250 ms intervals before (spontaneous), and after, olfactory nerve stimulation (evoked) under control conditions (red) and 20–30 min post-induction (black). *p<0.05. Error bars, mean±SEM.

We next asked whether disinhibition of MCs from the local glomerular inhibitory network would by itself lead to MC potentiation when paired with TBS of the ON. Gabazine, a GABA_A_ receptor antagonist, was locally puffed to the glomerular layer at two concentrations. A low concentration (2 µM) of gabazine resulted in a change in the MC firing pattern, including a slight to moderate increase in evoked spikes in a burst-like firing pattern (Figure 4*A* *1*). A higher concentration (10 µM) of gabazine exhibited a similar pattern of action but in some cells, especially during prolonged application, resulted in the silencing of MC spikes (data not shown). Extensive disinhibition of MCs by high dose gabazine may lead to a “seizure” effect on MC firing with cells showing a depolarization block. Interestingly, only the low concentration of gabazine led to a potentiation of MC evoked spikes (Figure 4*A* *1–3, D*) and that potentiation was only significant in the 0–50 ms AMPA period (pre-induction: 18.67±3.71 Hz; post-induction: 25±4.63 Hz; *n* = 6; *t* = 3.08, *p* = 0.027), whereas the higher concentration of gabazine did not affect the MC spiking pattern 30 min following TBS induction (*n* = 6; *t* = 0.450, *P* = 0.672; Figure 4*B,D*). We next showed that the 2 µM gabazine induced MC spike potentiation was NMDAR-dependent. Co-application of D-APV (500 µM) in the same puff pipette during TBS fully abolished the potentiating effect of 2 µM gabazine (pre-induction: 21.23±5.86 Hz; post-induction: 17.95±6.92 Hz; *n* = 5; *t* = 1.00; *p* = 0.372; Figure 4*C*1–3,*D*). This suggests that MC potentiation induced by pairing TBS and local glomerular disinhibition is also NMDAR mediated. Thus, disinhibition promoted by isoproterenol could contribute to an NMDA role in odor preference learning.

### Early Odor Preference Learning Activates Glomerular NMDA GluN1 Subunits During Learning Induction

**Figure 5:**
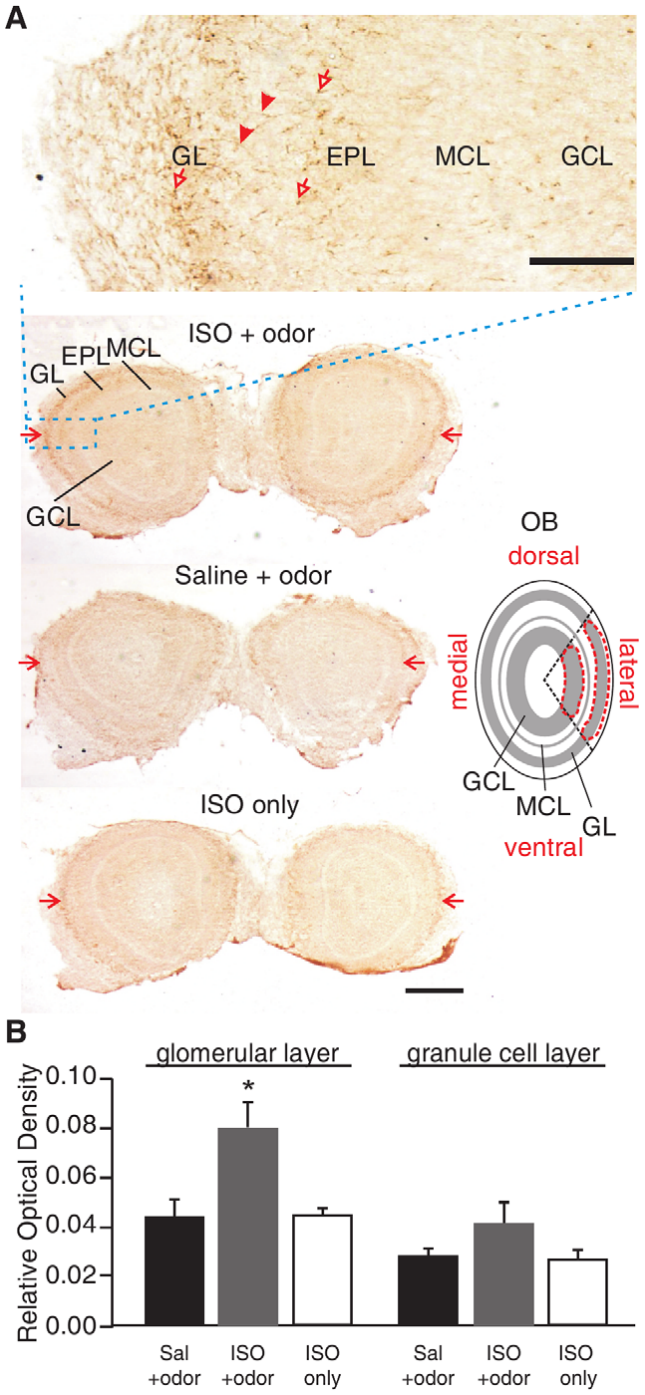
Early odor preference learning induced phosphorylation of the NMDA GluN1 (pGluN1) at 5 min following training. *A*. Immunohistochemistry of pGluN1 expression in the olfactory bulb 5 min following the end of training. Red arrows in the low magnification images indicate the mid-lateral glomerular layers where most significant changes were observed. Inset shows high magnification of a portion of the active region. Arrow heads indicate mitral cell processes. Hollow arrows indicate glial-like staining. Schematic on the right shows the region of interest for optical imaging analysis. GL, glomerular layer. EPL, external plexiform layer. MCL, mitral cell layer. GCL, granule cell layer. OB, olfactory bulb. Scale bars, 100 µm (for inset) and 500 µm. ***B*** **.** Analysis of relative optical density of pGluN1 staining in glomerular and granule cell layers 5 min post-training. Values presented are the relative optical density of the lateral glomerular and granule cell layers for pGluN1. *p<0.05. Error bars, mean±SEM.

To test the role of the NMDAR in our *in vivo* learning model, we first examined whether the NMDAR is activated following early odor preference learning and the localization of its activation in the olfactory bulb. It has been shown that phosphorylation of GluN1 affects the kinetics of the NMDAR, resulting in a larger current and greater calcium influx [30]. We used immunohistochemical staining with an antibody recognizing the PKA phosphorylation site (Ser897) of the obligatory GluN1 subunit of the NMDAR (pGluN1). We discovered that pairing the β-adrenoceptor agonist isoproterenol (2 mg/kg *s.c.*) with peppermint odor significantly increased pGluN1 expression in the mid-lateral portion of the glomerular layer of olfactory bulbs from pups sacrificed 5 min following odor training (*n* = 5 for ISO+odor group, 0.080±0.010; *n* = 4 for the control groups, 0.044±0.007 and 0.044±0.003 for saline+odor and ISO only groups; *F*_2,10_ = 6.79: *p* = 0.014; Figure 5B). In contrast, there was no difference in pGluN1 staining when the medial regions of the glomerular layer were compared (*F*_2,10_ = 1.54: *p* = 0.261). The location of pGluN1 activation is consistent with previous reports using a 2-DG tracing technique showing that peppermint odor activates glomerular “hot spots” in the mid-lateral portion of olfactory bulb glomeruli [6], [31], [32] and that these peppermint “hot spots” were enlarged in rat pups who underwent odor learning [9]. In the current study, immunohistochemistry revealed that pGluN1 staining in the glomerular layer was seen in processes (Figure 5A, arrow heads in the enlarged inset) and may correspond to dendritic structures in the glomeruli such as MC [33] and tufted cell dendrites. We also observed staining in small glial-like cells (Figure 5A, hollow arrows in the inset). We did not further pursue the identity of those cells but glial cells in the olfactory bulb express GluN1 [33] and glial activity (e.g. astrocytes) in the glomerulus mirrors that of MCs [34]. We also analyzed pGluN1 expression in the adjacent granule cell layer and found no significant changes in either the lateral (*F*_2,10_ = 1.60: *p* = 0.249; Figure 5*B*) or the medial region (*F*_2,10_ = 1.17: *p* = 0.35) among different experimental groups.

### β-adrenoceptor Mediated Early Odor Preference Learning is NMDAR-dependent

**Figure 6:**
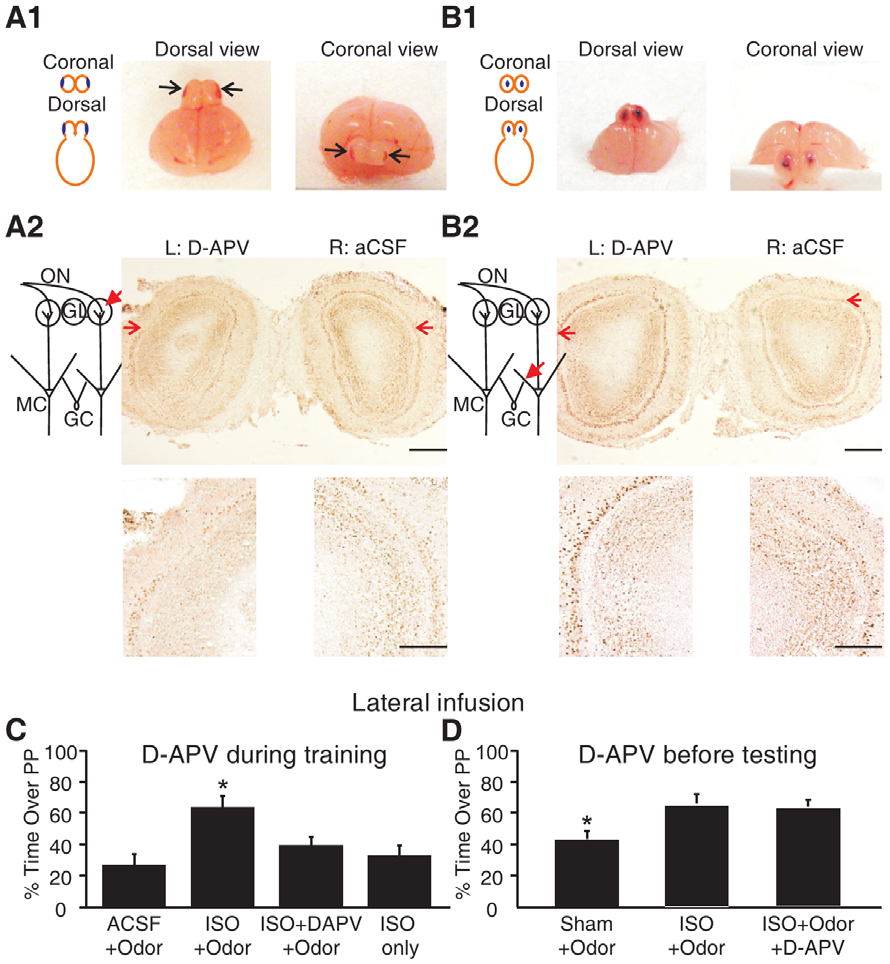
β-adrenoceptor mediated early odor preference learning was NMDAR-dependent. *A1-A2*. Lateral drug infusion site that selectively affects glomerular ON-MC synapses. ***A1*** **.** Mid-lateral infusion sites at either coronal or horizontal views of the olfactory bulbs, infused with methylene blue dye (4%). Black arrows indicate the sites of infusions. ***A2*** **.** pCREB immunohistochemistry staining shows that MC staining on the D-APV infusion site were ***suppressed*** compared to the control ACSF site, suggesting D-APV mainly acts on the ON-MC synapses in this preparation. Red arrows in the upper panel indicate the difference in pCREB staining in the lateral regions of the MC layers. Lower panel shows enlarged lateral regions of MC layers. Scale bars, 500 µm (upper panel) and 200 µm (lower panel). ***B1*** **.** Central infusion sites at either coronal or horizontal views of the olfactory bulbs, ***B2*** **.** pCREB immunohistochemistry staining shows that MC staining on the D-APV infusion sites was ***enhanced*** compared to the control ACSF sites, suggesting D-APV mainly acts on the GC-MC synapses in this preparation. Same labeling and enlargement were used as in *A2*. ***C*** **.** Early odor preference learning is blocked when an NMDAR antagonist D-APV is infused during training. Bars show the percentage of time spent in the peppermint side of a two-choice test box across different experimental groups. *p<0.05. Error bars, mean±SEM. ***D.*** D-APV infusion 10 min before testing does not prevent the odor preference formed with ISO+Odor pairing. *p<0.05. Error bars, mean±SEM.

We next explored a potential causal role of NMDAR activation in mediating odor preference learning. We directly infused isoproterenol (50 µM) into the olfactory bulbs of rat pups during odor training [7] to induce odor preference learning and tested whether co-application of D-APV (500 µM), a NMDAR antagonist, would block learning. We established a method that allowed us to infuse the drug mainly into the superficial layer of the olfactory bulb on the lateral surface, where enhanced pGluN1 expression was observed following odor preference learning (Figure 6*A* *1*). NMDAR is one of the excitatory synaptic transmission receptors at ON-MC synapses [35], [36], as well as mediating synaptic transmission from MCs to granule cells [37]–[39]. Depending on the synaptic site, NMDAR blockade would have differential effects on MC excitation. Previous research suggests that the NMDAR augments a long-lasting depolarization of MCs to ON stimulation [36], [40], [41]. If D-APV acts at ON-MC synapses to block NMDARs on MCs, we expected reduced MC excitation. This hypothesis is supported by our electrophysiology data (Figure 2*A* *1–4*) showing that D-APV application reduced ON stimulation-evoked MC spikes in the olfactory bulb slice preparation. In contrast, if D-APV acts more ventrally to block NMDARs on granule cells, this would lead to reduced activity of granule cells and subsequent disinhibition of MCs [37]–[39]. We developed a lateral infusion protocol targeting the area of peppermint representation. By infusing D-APV into one olfactory bulb and aCSF into the other, we confirmed that our lateral infusion protocol mainly affected NMDARs at ON-MC synapses because pCREB, an acute neuronal activity marker, showed ***reduced*** expression on MCs in the mid-lateral portion of the olfactory bulb with D-APV infusion compared to that with aCSF infusion (see results in Figure 6*A* *2,* 0.85±0.05 (ratio of D-APV to aCSF); *n* = 6; *t* = 2.852; *p* = 0.036). This is in contrast with a significantly ***enhanced*** pCREB expression pattern observed after we infused D-APV into the center of the olfactory bulb (as shown in Figure 6*B* *1*), which most likely affected NMDARs on granule cells (Figure 6*B* *2*, 2.07±0.29 (ratio of D-APV to aCSF); *n* = 4, *t* = 3.731, *p* = 0.034). Using the lateral infusion method, we discovered that D-APV blocked the learning effect caused by isoproterenol infusion during odor training (Figure 6*C*). Figure 6*C* shows that infusion of isoproterenol into the olfactory bulb during peppermint odor exposure successfully induced odor preference in young pups (63.5±7.3%), whereas control vehicle infusion (32.9±6.4%) or isoproterenol infusion without odor exposure (27.2±6.8%) failed to produce an odor preference (*n* = 6; *F*_3,20_ = 6.136; *p* = 0.004). Co-application of D-APV with isoproterenol completely blocked the isoproterenol effect in inducing odor preference (39.4±5.1%, *n* = 6; *t* = 2.703; *p* = 0.024). Furthermore, infusion of D-APV 10 min before testing the next day did not affect the odor preference memory formed by pairing odor with isoproterenol infusion (Figure *6D*; Sham+Odor: 43.3±5.3%, *n* = 7; ISO+Odor: 65.8±5.7%, *n* = 10; ISO+Odor+D-APV: 63.6±5.1%, *n* = 8; *F_2,24_* = 4.647, *p* = 0.021). This suggests NMDA receptors are critical in initiating learning at the glomerular level. It also argues that AMPA receptors, but not NMDA receptors, play a critical role in odor perception since rat pups with NMDA blockade during testing, but not training, behaved identically to those tested under ACSF infusion. Thus the preference established with drug-free training was unaltered by the NMDA blocker D-APV.

We also tested the effect of D-APV central infusions on learning. The D-APV centrally-infused pups showed odor preference learning compared to the control aCSF infused pups (Figure S1), suggesting granule cell disinhibition of MCs is sufficient to produce a learning signal.

### Glomerular Disinhibition Plays a Role in Early Odor Preference Learning

**Figure 7:**
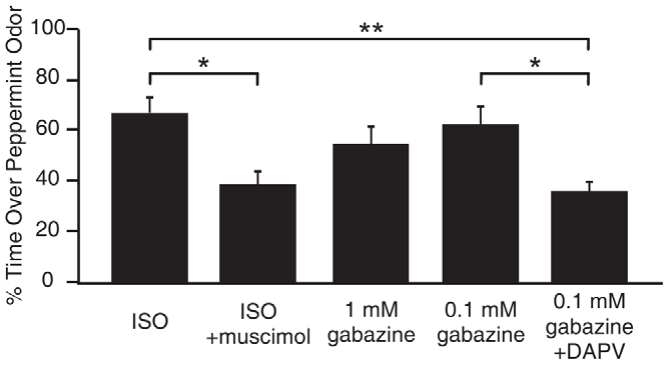
Glomerular disinhibition mimicked isoproterenol effect in inducing early odor preference learning. Bars show the percentages of time spent in the peppermint side in a two-choice test box in different experimental groups. **p<0.01 *p<0.05. Error bars, mean±SEM.

From our *in vitro* slice physiology data, we proposed that β-adrenoceptor activation could promote long-term potentation by suppressing the glomerular inhibitory network. Transient disinhibition of MCs by β-adrenoceptor activation would lead to increased NMDAR activation and sufficient calcium influx for long-term plastic changes at these synapses. We tested whether glomerular disinhibition could be a sufficient stimulus for early odor preference learning (Figure 7). We used the same infusion protocol as in Figure 6. One-way ANOVA analysis showed significant drug effects (F_4,34_ = 5.666, *p* = 0.001). We infused a high dose of muscimol (10 mM) together with isoproterenol into the dorsal-lateral olfactory bulbs. Co-application of muscimol blocked the early odor preference learning that could be induced by isoproterenol infusion (isoproterenol+muscimol: 38.34±5.40% on peppermint; *n* = 7; isoproterenol infusion alone: 66.89±6.41%; *n* = 8; post-Tukey *p* = 0.018). We applied two concentrations of gabazine to the olfactory bulbs. The lower dose (0.1 mM) of gabazine infusion led to odor preference learning almost comparable to isoproterenol (62.21±7.09%; *n* = 8), while the higher dose (1 mM) had a more modest and variable effect in promoting learning (54.47±6.94%; *n* = 8). D-APV co-application to the olfactory bulbs with 0.1 mM gabazine, blocked the learning effect caused by gabazine infusion alone (35.8±3.89%; *n* = 9; post-Tukey: *p* = 0.020).

### Early Odor Preference Learning Down-regulates NMDAR Subunits

Our data provide strong evidence that NMDARs critically mediate β-adrenoceptor induced early odor preference learning in rats. The activation of NMDARs seems critical for learning induction. We next explored the expression levels of NMDAR subunits at the time of memory following early odor preference learning. Activity-dependent modifications of NMDAR subunit composition can alter receptor function, and subsequently have a significant impact on the properties of synaptic plasticity [20], [21], [42], [43].

We performed Western blot analysis of olfactory bulb synaptoneurosome samples collected at 3 h or 24 h following early odor preference training. Synaptoneurosomes are membrane protein extractions enriched for synaptic proteins [21]. Previous studies demonstrated that this method greatly enhances the ability to detect synaptic NMDARs and other synaptic receptor proteins [44]. Immunoblotting was performed for NMDAR GluN1 and GluN2B subunits.

**Figure 8:**
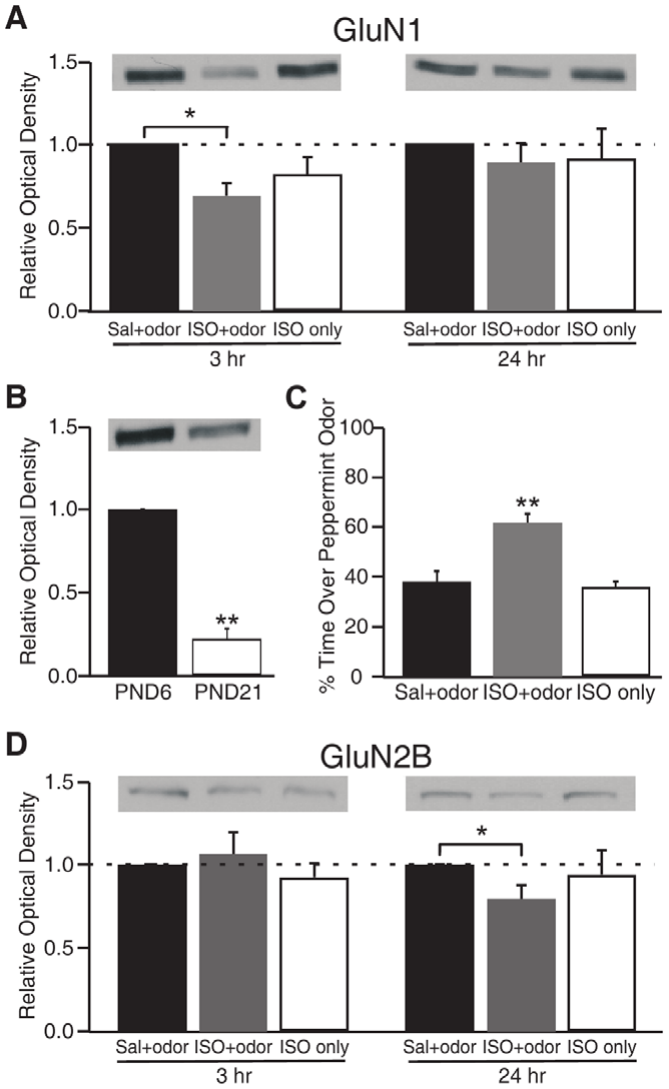
Early odor preference learning significantly down-regulated synaptic NMDAR subunits expression in the olfactory bulb. ***A*** **.** Western blot analysis of olfactory bulb synaptoneurosome samples collected at 3 h and 24 h following early odor preference training. Relative optical density values are normalized to the saline+odor group. ***B*** **.** Western blot analysis of olfactory bulb synaptoneurosome samples collected from naïve PND 6 and 21 rats. ***C*** **.** Two-choice odor test of the littermates. ***D.*** GluN2B expressions at 3 h and 24 h following early odor preference training. Relative optical density values are normalized to the saline+odor group. **p<0.01. *p<0.05. Error bars, mean±SEM.

Our analysis revealed that at 3 h following training, animals in the learning group (ISO+odor) showed significantly less expression of the obligatory GluN1 subunit of the NMDAR at synaptic sites within the olfactory bulb compared to control saline groups (Normalized to control, ISO+odor: 69.4±8.0%; ISO only: 82.3±10.1%; *n* = 16; *F*_2,45_ = 4.223, *p* = 0.021; Figure 8*A*). However, there was no significant difference in GluN1 expression 24 h following training (ISO+odor: 88.9±12.4%; ISO only: 91.4±17.9%; *n* = 15; *F*_2,42_ = 0.216, *p* = 0.807; Figure 8*A*), suggesting a transient and reversible change in GluN1 uisng our odor training protocol. Overall, the developmental pattern for the GluN1 subunit is down-regulation (PND 21, normalized to PND 6: 0.22±0.07; *n* = 5; *t* = 11.691; *p*<0.001; Figure 8*B*) suggesting the transient change at PND 6 may provide a window of reduced plasticity that helps stabilize learning. As predicted, littermates who received isoproterenol paired with peppermint odor exposure showed odor preference memory the next day, spending significantly more time over peppermint bedding than animals in control groups (ISO+odor: 61.6±4.0%; saline+odor: 38.1±4.0%; ISO only: 35.5±2.7%; *n* = 23; *F*_2,64_ = 15.743, *p*<0.001; Figure 8*C*).

These data strongly suggest that local glomerular disinhibition can mimic isoproterenol in inducing early odor preference learning and NMDA receptor activity is still required for this learning to occur.

**Figure 9:**
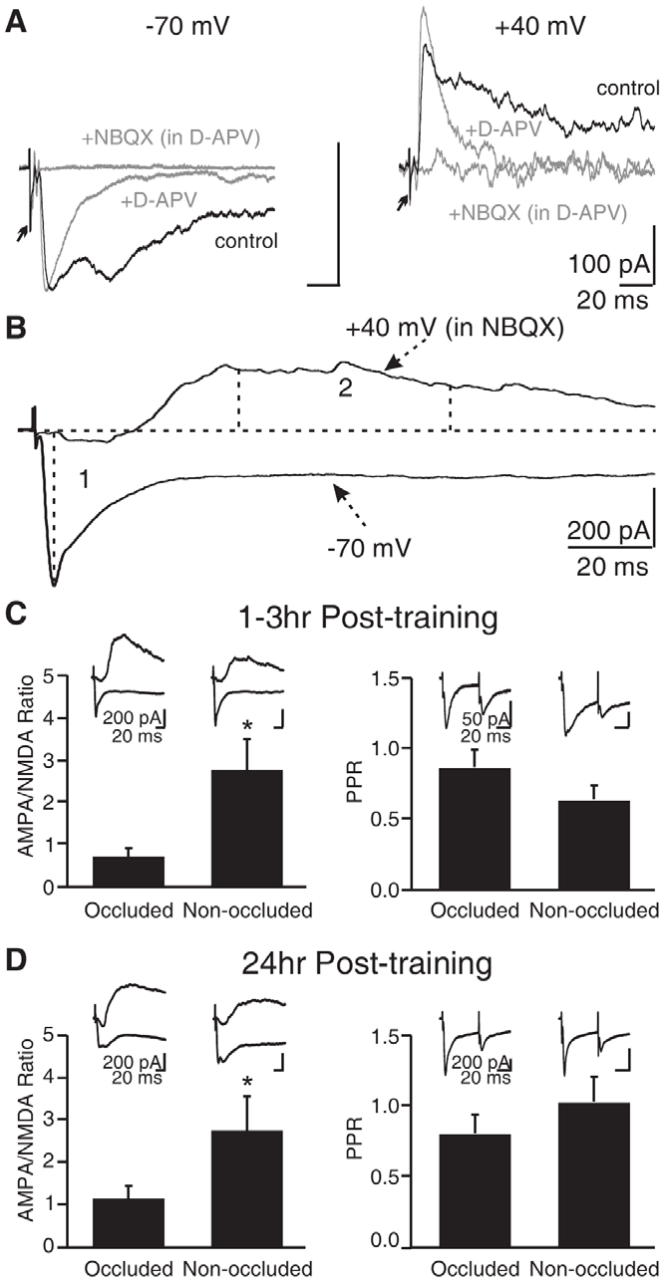
Early odor preference learning altered the ratio of MC AMPA/NMDA currents and the PPR in the olfactory bulb slices. ***A.*** Pharmacological isolation of AMPA and NMDA EPSCs at −70 mV and +40 mV in one example cell. Arrows indicate truncated stimulation artifacts. ***B.*** An example cell showing the measurement of the AMPA component (1, peak at −70 mV) and the NMDA component (2, average of 50–100 ms following the stimulation at +40 mV). ***C.*** The AMPA/NMDA ratio and PPR 1–3 h post-training. ***D.*** The AMPA/NMDA ratio and PPR 24 h post-training. *p<0.05. Error bars, mean±SEM.

The NMDAR consists of two obligatory GluN1 and two regulatory GluN2 subunits – either GluN2A or GluN2B [45]. Down-regulation of the GluN1 subunit specifically reflects the overall expression level of the NMDAR, while the composition of GluN2 subunits can undergo activity-dependent changes that alter receptor function [21], [45], [46]. For example, the GluN2B subunit confers slower and broader kinetics to the NMDAR, with opening characteristics that permit a bigger calcium influx [21]. Therefore, more GluN2B expression often favors greater synaptic plasticity [20], [21], [42], [43]. We examined GluN2B subunit expression following early odor preference learning at the same time points as GluN1 (Figure 9). Our analysis showed that not at 3 h (ISO+odor: 106.5±13.0% normalized to Saline group; ISO only: 92.3±9.1%; *n* = 19; *F*_2,54_ = 0.609, *p* = 0.547), but at 24 h following odor training, animals in the learning group (ISO+odor) show significantly less expression of the GluN2B subunit of the NMDAR at synaptic sites within the olfactory bulb compared to the saline group (ISO+odor: 79.3±8.5% normalized to Saline group; *n* = 19; *t* = 2.43, *p* = 0.026; Figure 8*D*). This is another change which would reduce plasticity and could act to stabilize NMDAR-dependent learning.

### Early Odor Preference Learning Significantly Alters the AMPA/NMDA Ratio of ON-evoked MC EPSCs

A recent report demonstrated AMPA GluA1 subunit up-regulation in the glomerular layer [47] following early odor preference learning. AMPAR insertion into “silent” NMDAR only synapses and the down-regulation of NMDAR subunits observed in our study fits well with activity-dependent changes of synaptic receptor trafficking. We next sought to understand the cellular locus of these receptor changes. We carried out whole cell recording experiments of MCs within the mid-lateral olfactory bulb at different time points post-training. From olfactory bulb slices of unilateral nasal occluded animals (10 min nasal occlusion during odor training, see Methods), we measured the AMPA/NMDA ratio of ON-evoked EPSCs from MCs of occluded (control) and non-occluded (learning) olfactory bulbs. At 1–3 h post-training, the AMPA/NMDA ratio of ON-evoked MC EPSCs from non-occluded olfactory bulbs was significantly higher than that recorded from occluded olfactory bulbs (non-occluded: 2.81±0.72, *n* = 12; occluded: 0.74±0.20, *n* = 10; *t* = 2.54, *p* = 0.010; Figure 9*C*). The AMPA/NMDA ratio at 24 h post-training showed a similar trend (non-occluded: 2.81±0.77, *n* = 10; occluded: 1.18±0.30, *n* = 8; *t* = 1.80, *p* = 0.045; Figure 9*D*). We also compared the AMPA/NMDA ratios of naïve slices from animals at the same ages with those of occluded ones and found no difference among these groups (data not shown), suggesting no effect of isoproterenol injection or acute nasal occlusion on the AMPA/NMDA ratios.

In order to test whether presynaptic changes are involved following early odor preference learning, the PPRs of peak EPSCs measured at −70 mV were compared between the two groups. Although not significant, the PPR of the non-occluded group was lower than that of the occluded group at 1–3 h post-training (non-occluded: 0.65±0.1, *n* = 12; occluded: 0.88±0.12, *n* = 10; *t* = 1.48, *p* = 0.077; Figure 9*C*). Interestingly, when cells recorded at 1 h post-training were examined alone, a clear difference between occluded and non-occluded cells was evident (non-occluded: 0.63±0.13, *n* = 7; occluded: 1.05±0.17, *n* = 4; *t* = 1.94, *p* = 0.042), suggesting there may be a transient presynaptic change involved in the early stages of odor preference memory formation. In line with this idea, there was no difference between the PPRs recorded from cells from non-occluded and occluded olfactory bulbs at 24 h post-training (non-occluded: 1.04±0.18, *n* = 9; occluded: 0.82±0.14, *n* = 7; *t* = 0.94, *p* = 0.182; Figure 9*D*).

## Discussion

### An Enhanced MC Excitation Model for Early Odor Preference Learning

McLean et al. proposed that the substrate for early odor preference learning in the olfactory bulb is enhanced excitation of MCs [2], [4], [19]. Enhanced CREB phosphorylation in MCs in odor-encoding “hot spots” has been observed following odor preference learning [2], and evidence suggests β-adrenoceptor promotion of the cAMP/PKA/CREB cascade in MCs of the olfactory bulb underpins memory formation [1]–[4]. The critical learning change is hypothesized to be a long-term facilitation of ON-MC synaptic transmission [4]. Increased excitation of odor-encoding MCs results in enhanced lateral inhibition to surrounding MCs, which sharpens the MC signals induced by the learned odor [4], [48]. The increased MC calcium responses observed 30 min after pairing ON-TBS and isoproterenol *in vitro* [19], and the enhanced MC CREB phosphorylation observed *in vivo* [2], support the MC excitation model. Other studies of the MC to granule cell synapse in the carp using antidromic activation of MCs have also shown LTP changes in MC activation of granule cells following activation of the cAMP cascade either directly or *via* 20 µM norepinephrine [49]. In an *in vivo* study of the carp these LTP changes were shown to associate with increased odor responses consistent with enhanced ON-MC activity [50]. Schoppa’s group also showed that during TBS-ON stimulation, norepinephrine induces a long-term increase in gamma frequency (30–70 Hz) synchronized oscillations recorded in the external plexiform layer [41]. This enhancement appears to be caused by increased excitatory drive on the mitral/granule cell network. Taken together, these results suggest the primary effect of an unconditioned stimulus, such as norepinephrine, is long-lasting potentiation of MC excitation.

Here, we directly tested the MC excitation hypothesis by measuring MC spike activity in acute olfactory bulb slices. Our results show that TBS of the ON, in conjunction with bath application of the β-adrenoceptor agonist isoproterenol, significantly increased MC-evoked spikes 30 min after the pairing induction. Neither TBS nor isoproterenol alone induced the long-term changes in evoked MC spiking activity seen with pairing. This result provides direct evidence that in a situation mimicking odor conditioned learning, long-lasting potentiation of MC responses can be induced. Further, we observed an increased ratio of AMPAR/NMDAR-mediated synaptic currents in the MCs of olfactory bulbs that underwent natural learning. A transient reduction in the PPR suggests an early presynaptic change may also be involved.

### Synaptic Mechanisms Underlying Enhanced MC Excitation – Glomerular Disinhibition

We were particularly interested in what the specific effects of β-adrenoceptor activation on MCs are that could lead to long-lasting synaptic potentiation. Studies using immunohistochemistry and receptor autoradiography show that β-adrenoceptors are expressed in MCs [4], juxtaglomerular cells [4], [51], and granule cells [51]. β-adrenoceptor expression on MCs raises the possibility that β-adrenoceptor activation acts directly to increase MC excitability. On average, we observed that acute application of isoproterenol increased spontaneous MC spikes. However, isoproterenol appears not to have a direct effect on MC excitability [28]. Although isoproterenol caused an inward current in MCs in voltage clamp, this inward current was abolished by synaptic transmission blockers, suggesting an indirect circuitry effect. Thus, isoproterenol may enhance MC excitation through disinhibition of either granule or periglomerular cells. In particular, isoproterenol suppresses evoked EPSCs and calcium responses in periglomerular cells [19]. This leads to the hypothesis that isoproterenol can disinhibit MCs through suppression of inhibitory glomerular neurons. Periglomerular neurons exert a powerful inhibitory output in the glomerulus, where they receive ON input and subsequently “gate” MC activities associated with the glomerulus [29]. Periglomerular feedforward inhibition suppresses MC long-lasting depolarization and preferentially filters MC responses to weak odor signals [29]. Disinhibition of MCs by inhibiting periglomerular neurons could enhance MC excitation and permit the level of calcium entry required for synaptic plasticity. Our data show that enhancing glomerular inhibition by muscimol puffs to the glomerular layer prevents the potentiation of MC spikes induced by the pairing of TBS and isoproterenol. Furthermore, local, as indicated by phenol red, glomerular disinhibition by a low dose of gabazine, when paired with TBS, results in MC spike potentiation in the absence of isoproterenol. Further tests of the effect of isoproterenol on directly connected PG and MCs using paired electrical recording or optical imaging would provide more direct evidence regarding the role of isoproterenol and periglomerular disinhibtion.

Given these effects on ON-MC plasticity, we employed an infusion method to study the effect of glomerular disinhibition on learning. We reasoned that if isoproterenol causes depolarization of MCs through glomerular disinhibition, then enhancing glomerular inhibition by muscimol infusion would prevent isoproterenol-mediated early odor preference learning. Our results show that co-infusion of muscimol completely blocked isoproterenol-induced learning; however this could have occurred due to loss of odor signaling at encoding. We tested this possibility by examining the effect of muscimol on normal peppermint aversions. The same infusion of muscimol prevented the normal peppermint aversion seen without training (Figure S2) and suggests that odor signaling is altered by muscimol. More convincingly in support of an important role for disinhibition, our data showed that a glomerular infusion of the GABA_A_ antagonist, gabazine, paired with odor, produced an odor preference. We infer that local disinhibition in the glomeruli responding to peppermint is sufficient for odor preference learning. We cannot, of course, rule out in the *in vivo* infusions that there is some contribution of granule cell disinhibition. The work of Kaba demonstrated that manipulation of inhibition in the olfactory bulb by whole bulbar infusion of a GABA_A_ receptor agonist, or antagonist, blocked or induced odor learning in PND 12 rats [52]. In our preparation, the use of a lateral infusion method, which did not increase MC excitation as indicated in our pCREB test in the D-APV infusion experiment (Fig. 6), suggests glomerular disinhibition alone may be effective in inducing learning. This is supported by the *in vitro* plasticity data using gabazine puff application to the glomeruli. We propose that both beta-adrenergic-mediated disinhibition [19] and phosphorylation of mitral cell GluN1 subunits [47] act in concert to enhance NMDA calcium currents and promote local olfactory nerve-mitral cell potentiation.

### Critical Role of NMDAR in Early Odor Preference Learning

The NMDAR, common in dendritic structures of learning-type neurons, has long been considered to be a critical mediator of associative plasticity [53]. NMDARs are located on both the apical and lateral MC dendrites [35], [54] and mediate a component of ON-evoked MC synaptic potentials [35], [36]. NMDARs on MCs are ideal as coincidence detectors, to associate the presynaptic glutamate release triggered by ON input, with the postsynaptic MC depolarization provided by isoproterenol disinhibition. We found that D-APV co-application with isoproterenol blocked MC spike potentiation *in vitro* and odor preference learning *in vivo*. The causal effect of NMDAR activation in learning induction was complimented by a correlational increase of GluN1 phosphorylation in the learning group (ISO+odor) shortly following odor training. The locus of pGluN1 change appears to be in the glomerular layer corresponding to the peppermint-responsive region.

In our study, D-APV modulation of MC spiking was consistent with intracellular MC recordings showing that D-APV blocks a late component of EPSCs [35], [55]. The AMPA receptor mediates fast and short-latency spikes within 50 ms of stimulation onset [27]. Since D-APV application suppressed MC-evoked spikes, there is a possibility that D-APV could affect odor perception. However, D-APV infusion before testing did not change the odor preference formed by pairing isoproterenol with odor. This result rules out the possibility that local NMDAR blockade affects odor perception (in that case, animals would spend equal amounts of time on peppermint and control bedding) or memory retrieval (in that case, animals would spend less time on peppermint similar to control non-learning pups). D-APV blockade of both gabazine-induced MC spike potentiation as well as early gabazine-induced odor preference learning, raises the possibility that β-adrenoceptor activation and glomerular disinhibition share common mechanisms in the induction of odor preference learning.

### NMDAR Down-regulation Following Early Odor Preference Learning

While the NMDAR is activated during learning acquisition, we found a down-regulation of NMDAR responses during the memory phase. These were of two types. Within a memory interval that does not depend on protein synthesis [56], that is 3 h post odor training, GluN1 obligatory subunits significantly decreased in the synaptosomal preparation suggesting less NMDA receptor insertion or more NMDA receptor degradation. AMPA receptor insertion appears to increase by 3 h post training in the same paradigm [47]. At the time of 24 h memory, which is dependent on protein synthesis, GluN1 measurements suggest NMDA receptor insertion and/or degradation has returned to baseline (AMPA receptor insertion remains elevated at this time point [47]). However, the composition of the NMDA receptor complex is significantly altered with a reduction in the NR2B subunit. The NR2B subunit is associated with larger NMDA currents and greater synaptic plasticity [21]. Taken together these two events suggest plasticity is down-regulated following conditioning. Such functional down -regulation of the NMDAR may help to prevent further synaptic change and enable the consolidation of the newly-forming memory. Odor experience itself has been previously shown to reduce NMDA currents in the olfactory cortex when comparing pyramidal neurons in the piriform cortex deprived of input for days to those non-deprived [20]. In a single nostril odor preference training paradigm, we have recently demonstrated physiologically at 24 h post training that NMDA current is reduced and AMPA current is increased for olfactory nerve input to learning synapses [57], consistent with the molecular changes observed in Cui et al, 2011 [47] and in the present study. This suggests the change in AMPA/NMDA EPSC ratio seen in MCs in the present experiment is mediated by both increases in AMPA receptors and changes in NMDA receptor composition that reduce current flow. In contrast, initial NMDAR-mediated MC firing appears stronger in the first 30 min in the *in vitro* preparations that have undergone ‘training’, one would predict weaker NMDA responses at longer time intervals based on the membrane subunit data. Phosphorylation of NMDARs was reported earlier [47] to occur up to 30 min following training, and could underlie the increase in NMDA-driven responses seen in the first half hour. This would be consistent with the failure to see an increase in NMDA-driven responses in the gabazine model in which β-adrenoceptor mediated PKA phosphorylation of NMDARs would not be predicted.

NMDAR-dependent synaptic activity also regulates the composition and function of the NMDAR itself [21], [42]. With learning in adult rats, the GluN2B has previously been shown to down-regulate, replaced by GluN2A [44]. This is consistent with our finding of a reduction of GluN2B expression 24 h following odor training. However, the reduction of GluN2B at 24 h post-training compared to controls suggests a functional change in NMDARs with learning, in addition to the developmental critical period down-regulation in this system. It would be interesting to determine, whether in the 3–24 h time period after acquiring an odor preference, pups show resistance to changing their response to the learned odor and/or if they have an altered plasticity response when tested with odors that overlap in glomerular representation with the learned odor.

## Materials and Methods

### Animals and Ethics Statement

Sprague Dawley rat pups (Charles River) of both sexes were used in this study. Day of birth was considered to be postnatal day (PND) 0 and litters were culled to 12 pups on PND 1. Dams were maintained under a 12 h reverse light/dark cycle at 22°C in polycarbonate cages with *ad libitum* access to food and water. All experimental procedures were approved by the Institutional Animal Care Committee at Memorial University of Newfoundland (protocol number: 11–01-QY) and follow the guidelines set by the Canadian Council on Animal Care.

### In vitro Eletrophysisology

#### Slice preparation and extracellular recording

PND 7–13 rats were anesthetized with halothane inhalation and decapitated. The brains were dissected and placed into ice-cold artificial cerebrospinal fluid (aCSF) containing the following (in mM): 83 NaCl, 2.5 KCl, 0.5 CaCl_2_, 3.3 MgSO_4_, 1 NaH_2_PO_4_, 26.2 NaHCO_3_, 22 glucose, and 72 sucrose equilibrated with 95% O_2_ and 5% CO_2_. Horizontal olfactory bulb slices were cut at 400 µm using a vibrating slicer (Leica VT 1000P) and incubated at 34°C for 30 min in the same high glucose aCSF. Slices were then left at room temperature until use. During recording, slices were superfused with aCSF containing the following (in mM): 119 NaCl, 2.5 KCl, 2.5 CaCl_2_, 1.3 MgSO_4_, 1 NaH_2_PO_4_, 26.2 NaHCO_3_, 22 glucose equilibrated with 95% O_2_ and 5% CO_2_ and viewed with an upright microscope (Olympus BX51) using differential interference contrast (DIC) optics. Extracellular loose patch recordings were obtained with glass pipettes filled with aCSF (2–3 MΩ) and positioned at the cell body of MCs. The stimulation pipette was placed at the ON layer adjacent to the glomeruli that were innervated by the primary dendrites of the recorded MCs. The ON was stimulated by a single test stimulus (20–100 µA) every 20 sec using a concentric bipolar stimulating pipette (FHC). The intensity of the stimulation was adjusted to evoke non-saturating MC spikes (approximately 50–60% of the maximum spikes). Theta burst stimulation (TBS, 10 bursts of high frequency stimulation at 5 Hz, each burst containing five pulses at 100 Hz, with the same stimulation intensity as the test stimuli) that mimics the sniffing cycles in the ON [58] was given after a baseline was taken. Electrophysiological data were recorded with a Multiclamp 700B (Molecular Devices), filtered at 2 kHz and digitized at 10 kHz. Data acquisition and analysis were performed with pClamp10 (Molecular Devices) and Igor Pro 6.10A (WaveMetrics). All experiments were conducted at 30–32°C, and data are mean±SEM. Student’s *t*-test and one-way ANOVA were used to determine statistical significance.

#### Drug application

The β-adrenoceptor agonist isoproterenol (10 µM, Sigma-Aldrich) was bath applied in all experiments. The NMDAR antagonist D-APV (50 µM, Tocris) and the AMPA receptor antagonist NBQX (40 µM, Tocris) were used in bath application with the experiments in Figures 1 and 2. The GABAA receptor antagonist muscimol (0.2–10 µM, Tocris), the GABAA agonist gabazine (2 µM or 10 µM, Tocris) and D-APV (500 µM) were locally puffed in the glomerular layer of olfactory bulb slices (Figures 3B and 4). For local puffing of drugs on the glomerular layer adjacent to the stimulation pipette, a glass pipette (1–2 MΩ) was placed in the ON next to the glomerular layer. The glass pipette was filled with drugs dissolved in aCSF and drugs were puffed by pressure. The extension of the puff flow was monitored by a red dye, phenol red (0.1–1%) dissolved in the same pipette solution. Phenol red puff itself (in aCSF) had no effect on MC responses. Pressure was adjusted and controlled so that the red solution spread to 2–5 glomeruli surrounding the stimulation pipette.

### Behavioral Studies Overview

Behavioural conditioning and testing occurred in a temperature controlled room at approximately 28°C and followed the standard protocol previously established for early odor preference learning [59]. An initial study assessed PKA-mediated phosphorylation of the obligatory GluN1 subunit of the NMDAR immediately following training. Based on the results of this pGluN1 experiment and previous work using 2-DG [31], [32], we next developed a protocol for intrabulbar infusion that allowed us to specifically target peppermint odor regions in the lateral olfactory bulb. We examined MC pCREB levels in olfactory bulbs from pups sacrificed 10 min post training. This time point was chosen as it is associated with maximal training-induced pCREB activation. We compared the effectiveness of lateral and central cannulae placements for the infusion of D-APV in modifying pCREB expression. Specific details of this analysis are given under the pCREB immunohistochemistry analysis below. The lateral placements shown to be effective (Fig. 6A and 6B) were used in all subsequent intrabulbar behavioral experiments where we investigated the role of NMDA and GABAA receptors in odor preference learning. Finally, *ex vivo* experiments were carried out to examine olfactory bulb NMDAR changes following learning. Specifically, regulation of the GluN1 and GluN2B subunits of the NMDAR at 3 h and 24 h following odor preference training was examined in olfactory bulb synaptoneurosomes. Changes of the relative strengths of AMPAR/NMDAR mediated synaptic currents were also examined by MC recordings from olfactory bulb slices of pups trained with unilateral naris plugs. Student’s *t*-tests and one-way ANOVAs were used to determine statistical significance throughout the experiments.

### pGluN1 Immunohistochemistry

Animals underwent odor preference training where they were individually removed from the nest briefly to receive a subcutaneous injection of either saline or isoproterenol (2 mg/kg, made in saline;) [59], and then returned to the nest. Thirty min following injection, each pup was individually placed on unscented clean bedding for a 10 min habituation period before being transferred to peppermint scented bedding (0.3 ml peppermint extract per 500 ml clean bedding) for a 10 min odor exposure period. A third group received only an isoproterenol injection with no exposure to peppermint odor, remaining on unscented bedding for 20 min. At 5 min following the end of the training period, animals were deeply anesthetized with chloral hydrate (Sigma-Aldrich) and perfused transcardially with ice-cold saline solution followed by ice-cold fixative solution (4% paraformaldehyde in 0.1 M phosphate buffer, pH 7.4). Brains were removed from the skull with olfactory bulbs intact and post-fixed for 1 hr in the same solution, after which they were immersed in 20% sucrose solution overnight at 4°C. The next day, brains were quick-frozen in dry ice and 30 µm coronal sections were cut in a cryostat at −20°C. Sections from animals in each treatment group within the same experiment were mounted together on the same slide in order to ensure uniform staining development across experimental groups. The pGluN1 antibody (1∶500, Abcam) was used to probe for phosphorylation of the NMDAR at the Ser897 PKA-mediated phosphorylation site. The antibody was dissolved in phosphate buffered saline with 2% Triton-X-100, 0.002% sodium azide, and 5% normal goat serum and applied to sections overnight at 4°C in a humidified chamber. The next day, sections were incubated in a biotinylated secondary antibody (Vectastain Elite) followed by a diaminobenzidine tetrahydrochloride reaction. Sections were dehydrated and coverslipped with permount (Fisher Scientific).

#### Image analysis for pGluN1 immunohistochemistry

Staining for pGluN1 was analyzed using a Bioquant image analysis system (R&M Biometrics). Images of sections were captured with a CCD camera connected to a Leitz microscope. The light intensity of the microscope was kept at the same level for all sections analyzed. For each section analyzed, the optical density (OD) of the ON layer was used as a measure of background OD. After taking a captured image of a section, regions of interest (ROI) were selected using a hand tracing tool. The relative OD of each ROI was obtained using the following formula: (OD of ROI – OD of background)/OD of background. Image analysis was conducted on every 3^rd^–4^th^ section beginning from the most rostral extent of the olfactory bulb until the accessory olfactory bulb was reached caudally. For each section, regions analyzed included the lateral and medial portions of the glomerular layer, as well as the lateral and medial portions of the granule cell layer lying directly subjacent to those areas of the glomerular layer analyzed (outlined in Figure 5A, right panel). The relative ODs across the rostrocaudal extent measured were compared for the lateral and medial regions among groups. This was an attempt at specifically targeting training odor induced changes as previous studies have reported peppermint “hotspots” to be located on the lateral surface of the olfactory bulb [6], [31], [32]. Values reported are mean±SEM for each ROI measured.

### Cannulae Implantation Surgery and Drug Infusion

Two guide cannulae (Vita Needle Company Inc.; 23 gauge tubing cut to 6 mm) were anchored in dental acrylic (Lang Dental) such that they were separated laterally by approximately 4 mm and extended beyond the acrylic by approximately 0.5–1 mm. Insect pins were placed inside the guide cannulae to prevent blocking.

On PND 5 rat pups were anesthetized via hypothermia and placed in a stereotaxic apparatus with bregma and lambda in the same horizontal plane. The skull was exposed and the olfactory bulbs were visualized through the thin skull. Two small holes were drilled over the dorsal-lateral surface in the central plane of each olfactory bulb. The cannulae were lowered into the olfactory bulb and the assembly was fixed to the skull with dental acrylic. The skin was sutured together and pups were allowed to recover from anesthesia on warm bedding before being returned to the dam and littermates.

Infusion cannulae were made from 30 gauge stainless steel tubing (Small Parts Inc.) cut to a length of approximately 13 mm and inserted into PE20 polypropylene tubing (Intramedic). Each infusion cannula was inserted into a piece of tubing so that 7 mm of cannula extended beyond the end of the tubing. For bilateral olfactory bulb infusion, the other end of the tubing was secured over the needle of a 10 µl microsyringe (Hamilton Company). The two syringes attached to the infusion cannulae were placed in a multi-syringe pump (Chemyx). At infusion, the insect pins were removed from the guide cannulae and the infusion cannulae were gently inserted into the olfactory bulb through the guide cannulae assembly previously fixed to the animal’s skull.

### pCREB Immunocytochemistry for Checking Infusion-associated Changes in MC Activity

To establish the relationship between the placement of infusion cannulae and MC activity, pCREB immunocytochemistry was carried out. PND 6 rats were exposed to peppermint-scented bedding for 10 min. During the odor exposure period, aCSF was infused into one bulb and D-APV (500 µM made in aCSF) was infused into the other bulb at a rate of 0.1 µl/min for 10 min. Five to ten minutes following the end of odor exposure, animals were deeply anesthetized and perfused transcardially as described above. Section treatment was as for the pGluN1 experiment except that a pCREB antibody (1∶100, Cell Signalling) was used. In all animals, we observed lighter or similar MC pCREB staining when D-APV was infused laterally (compared with the aCSF control side) and significantly darker pCREB staining when D-APV was infused centrally, supporting the utility of selective cannulae placement. A quantitative assessment of placement differences was carried out by imaging analyses of the optical density of the mid-lateral MC layer using the same method as for the pGluN1. Six laterally infused and four centrally infused animals with optimal bilateral cannulae positioning and intact sections throughout the olfactory bulbs as well as optimal pCREB staining were used for this analysis. The ratios of the optical densities of the D-APV and the aCSF-infused olfactory bulbs from each animal were compared.

### Intrabulbar Infusion Experiments

In all subsequent experiments animals received the β-adrenoceptor agonist isoproterenol (50 µM), administered directly into the olfactory bulbs via intrabulbar infusion as the unconditioned stimulus [7]. All drugs for infusion were made in aCSF.

#### NMDAR antagonist experiment

During training on PND 6, animals received bilateral intrabulbar infusion of aCSF, isoproterenol, or isoproterenol together with D-APV (500 µM). Infusion occurred at a rate of 0.05 µl/min for 20 min over the course of the habituation period and the odor exposure period, the total volume infused was 1µl/bulb. The next day, pups were tested for odor preference memory as described below. To test whether D-APV infusion affects odor detection and memory acquisition, we performed another set of experiments with D-APV infusion 10 min before testing while isoproterenol was infused during training on PND6. A group of animals with sham surgery and 10 min odor exposure served as the non-learning control while another group with only isoproterenol infusion during odor training (no D-APV infusion before testing) served as a normal learning control.

#### GABAA receptor experiment

During training on PND 6, animals received bilateral intrabulbar infusion of either isoproterenol, isoproterenol together with muscimol (20 mM, Tocris), gabazine (0.1 mM, 1 mM, Tocris), or gabazine together with D-APV (0.5 mM). In this experiment again, a total volume of 1 µl per bulb was administered at a rate of 0.1 µl/min during the 10 min odor exposure period. The next day, pups were tested for odor preference memory as described below.

#### Two-choice odor testing

On PND 7 each pup was tested for odor preference memory. A stainless steel box (30×20×18 cm) was placed on top of two training boxes separated by a 2 cm neutral zone. One box contained peppermint scented bedding while the other box contained clean, unscented bedding. Each pup was removed from the nest and underwent five separate 1 min trials during which they were placed in the neutral zone of the test box and allowed to move freely. After each trial the pup was removed from the test box for a 1 min intertrial interval. During testing, when the pup’s nose moved from the neutral zone to either the peppermint side or the unscented side, the experimenter began recording time. The total amount of time spent over peppermint scented bedding and unscented bedding over all 5 trials was calculated separately. Values reported are the percentages of time animals spent over the peppermint scented bedding divided by the total time spent over peppermint+unscented bedding combined. This follows the standard protocol pioneered by Sullivan et al [13]. Typically rat pups dislike peppermint odor. Naïve and control animals usually spend only 20–40% of time over peppermint scented bedding [13], [18], suggesting a natural aversion to the odor. Odor preference is demonstrated by pups trained under normal pairing protocols spending a significantly higher proportion of total time over peppermint scented bedding compared to pups from non-learning control conditions.

#### Cannulae placement verification

After testing, those animals who had received intrabulbar infusions during training received a final intrabulbar infusion of methylene blue dye (4%, Fisher Scientific) at the same rate and volume as during training. Following infusion, pups were sacrificed and the olfactory bulbs were examined to ensure correct placement of cannulae in the glomerular layer of the dorsal-lateral olfactory bulb. Pups with incorrect cannulae placements were excluded from analysis.

### Western Blots on Synaptoneurosomes

#### Behavioral procedure and sample collection

Animals received a subcutaneous injection of either saline or isoproterenol (2 mg/kg) and underwent odor preference conditioning, while a separate group received isoproterenol alone without odor exposure as previously described. Following training, pups were returned to the nest. At 3 h or 24 h following training, animals were sacrificed and olfactory bulbs were rapidly removed and flash frozen on dry ice. All samples were stored at −80°C until use. Littermates were tested at 24 h for odor preference learning as described earlier.

#### Synaptoneurosome isolation

Isolation of synaptoneurosomes (a protein extraction enriched with synaptic protein) was performed as described elsewhere [60], [61]. Briefly, whole olfactory bulbs were homogenized using Teflon-glass tissue homogenizers (Thomas Scientific). Samples were homogenized in ice-cold HEPES buffer containing (in mM): 50 HEPES, 124 NaCl, 26 NaHCO_3_, 1.3 MgC_l2_, 2.5 CaC_l2_, 3.2 KCl. 1.06 KH_2_PO_4_, 10 glucose, 1 EDTA, 1 PMSF, complete protease inhibitor cocktail (Roche), complete phosphatase inhibitor cocktail (Roche), and saturated with 95% O_2_/5% CO_2_ (pH 7.4). Following a 10 min incubation period on ice, homogenates were passed through a series of filters held in syringe filter holders (Millipore); first through two 100 µm nylon filters (Small Parts Inc.), then through a 5 µm filter (Millipore). Next, the filtrate was centrifuged at 1000×g for 20 min at 4°C. After centrifugation, the synaptoneurosome pellet was resuspended in ice-cold HEPES buffer and protein concentrations were determined using a BCA protein assay kit (Pierce). Samples, standards and reagents were added to a 96 well plate and incubated at 37°C for 30 min. Next, the plate was read at 540 nm on a BIO-RAD Model 3550 Microplate Reader. The concentration of protein in each sample was calculated using a standard curve generated from values of standards run on the same plate. The volume of lysate required to obtain 40 µg of protein for each sample was determined according to the calculated protein concentrations of each sample.

#### Western blot

Sample solutions were prepared using 4 µl of 5X sample buffer (0.3 M TRIS-HCl, 10% SDS, 50% glycerol, 0.25% bromophenol blue, 0.5 M dithiothreitol), lysate (volume determined to contain 40 µg protein), and enough dH2O to bring the total volume to 20 µl. Sample solutions were then boiled for 5 min before being loaded into lanes of a 7.5% SDS-PAGE gel. Samples were separated by SDS-PAGE and then transferred to a nitrocellulose membrane (Amersham). After transfer was complete, membranes were cut horizontally at the 72 kDa level; the top portion was probed with an antibody recognizing either GluN1 (1∶1000, Cell Signalling) or GluN2B (1∶1000, Millipore), while the bottom portion of the membrane was probed with an antibody recognizing β-actin (1∶2000, Cedarlane). Membranes were immersed and agitated in primary antibody overnight at 4°C. The next day, antibodies were detected using a horseradish peroxidase-conjugated secondary antibody (Pierce), visualized with Super West Pico Chemiluminescent Substrate (Pierce), and developed on X-ray film (AGFA). Samples collected from the same litter and within the same experiment were processed together. Using an image scanner (CanoScan LiDE 200), blots were scanned and the optical density of each band was measured using ImageJ software. The optical density of the band of interest for each sample was normalized to the optical density of the β-actin band for that sample run on the same gel. Next, for each experiment this value was normalized to that of control animals (saline+odor) to determine differences in expression compared to non-learning littermates.

### Ex vivo Whole Cell Electrophysiology Experiments

On PND 6–9 animals underwent odor preference training with a subcutaneous injection of 2 mg/kg isoproterenol as described earlier, except that unilateral nasal occlusion was performed immediately before training by applying an odourless silicone grease plug to one nostril. At the end of the odor exposure period, the grease plug was removed from the occluded nostril and pups were returned to the nest.

#### Slice preparation and electrophysiology

At either 1–3 h or 24 h following odor preference training, pups were anaesthetized via halothane inhalation and decapitated. Horizontal olfactory bulb slices were prepared as described earlier in *in vitro* electrophysiology, except that slices were hemisected and those from occluded and non-occluded olfactory bulbs were separated before incubation at 34°C for 30 min in the same high glucose aCSF. Slices were then left at room temperature until use. During recording, slices were superfused with a Mg^2+^ free aCSF containing the following (in mM): 122 NaCl, 2.5 KCl, 2.5 CaCl_2_, 1 NaH_2_PO_4_, 26.2 NaHCO_3_, 22 glucose and equilibrated with 95% O_2_/5% CO_2_. Whole cell patch recordings were obtained using glass pipettes (2–6 MΩ) filled with an internal recording solution containing the following (in mM): 123 K-gluconate, 2 MgCl_2_, 8 KCl, 0.2 EGTA, 10 HEPES, 4 Na_2_-ATP, 0.3 Na-GTP, pH 7.35. Recording pipettes were positioned at the cell body of MCs within the mid-lateral olfactory bulb whose primary dendrites could be followed to the glomerular layer. The stimulation configuration was the same as in the earlier *in vitro* experiments. The intensity of the stimulation was adjusted to evoke a MC response when the cell was held in voltage clamp at both –70 mV and +40 mV. Electrophysiological data were recorded with Multiclamp 700B (Molecular Devices), filtered at 2 kHz and digitized at 10 kHz. Data acquisition and analysis were performed with pClamp10 (Molecular Devices) and Igor Pro 6.10A (WaveMetrics). All experiments were conducted at 30–32°C. The membrane resistance and access resistance for each cell was monitored throughout each experiment. All cells had an access resistance between 10–25 MΩ and any cells whose access resistance changed >30% during recording were discarded.

#### AMPA/NMDA EPSC ratio

The AMPAR and NMDAR mediated components of ON-evoked MC EPSCs were dissociated and measured during recording (see Figure 9*A, B*). The AMPAR component of a MC EPSC was recorded when the cell was held at –70 mV and consisted of a large negative peak immediately following ON stimulation. The NMDAR component of a MC EPSC was recorded when the cell was held at +40 mV in the presence of the AMPAR antagonist NBQX (20 µM). This NMDA-mediated EPSC consisted of a slower, longer lasting positive current measured between 50–100 ms following ON stimulation. The ratio of the AMPAR and NMDAR components of MC EPSCs were measured to obtain an AMPA/NMDA ratio for each cell. Values reported are mean±SEM of the AMPA/NMDA ratio for occluded (control) and non-occluded (learning) slices.

#### Paired-pulse ratio

To examine whether early odor preference learning modifies presynaptic release, the paired-pulse ratio (PPR) of two evoked EPSCs with an inter-stimulation interval of 50 ms was measured while the cell was held in voltage clamp mode at –70 mV. A PPR of ON-evoked MC EPSCs for each cell was calculated (ratio of EPSC2/EPSC1). Values presented are the mean±SEM for occluded (control) and non-occluded (learning) slices.

## Supporting Information

Figure S1 **D-APV central bulbar infusion induced odor preference learning in rat pups.** D-APV (50 µM, 1 µl; N = 6) or vehicle aCSF (N = 6) was infused centrally in the bilateral olfactory bulbs during odor training. The pups were tested for odor preference 24 hr later. D-APV central infusion induced odor preference when compared to the control (*t* = 2.335, *p* = 0.021). Bars show the percentages of time spent on the peppermint side in a two-choice test box in different experimental groups. *p<0.05. Error bars, mean±SEM.
(TIF)

Figure S2 **Muscimol lateral bulbar infusion interfered with peppermint odor perception in rat pups.** Muscimol (10 mM, 1 µl; N = 6) or vehicle aCSF (N = 6) was infused laterally in the olfactory bulbs. The pups were tested for odor preference 10 min after the infusions. Muscimol-infused pups lost the natural aversive response to peppermint bedding which was shown by the control pups (*t* = 2.227, *p* = 0.025). Bars show the percentages of time spent on the peppermint side in a two-choice test box in the two experimental groups. *p<0.05. Error bars, mean±SEM.
(TIF)
