# Supplementary figures and images for: Olfactory Bulb Glomerular NMDA Receptors Mediate Olfactory Nerve Potentiation and Odor Preference Learning in the Neonate Rat
Source: PLoS One. 2012 Apr 4;7(4):e35024. doi: 10.1371/journal.pone.0035024 (PMC3319620; doi:10.1371/journal.pone.0035024)

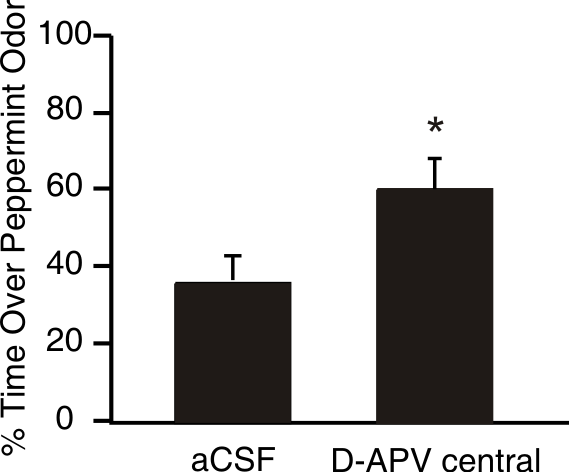

Supplement: Figure S1 — D-APV central bulbar infusion induced odor preference learning in rat pups. D-APV (50 µM, 1 µl; N = 6) or vehicle aCSF (N = 6) was infused centrally in the bilateral olfactory bulbs during odor training. The pups were tested for odor preference 24 hr later. D-APV central infusion induced odor preference when compared to the control (t = 2.335, p = 0.021). Bars show the percentages of time spent on the peppermint side in a two-choice test box in different experimental groups. *p<0.05. Error bars, mean±SEM. (TIF) [file pone.0035024.s001.tif]

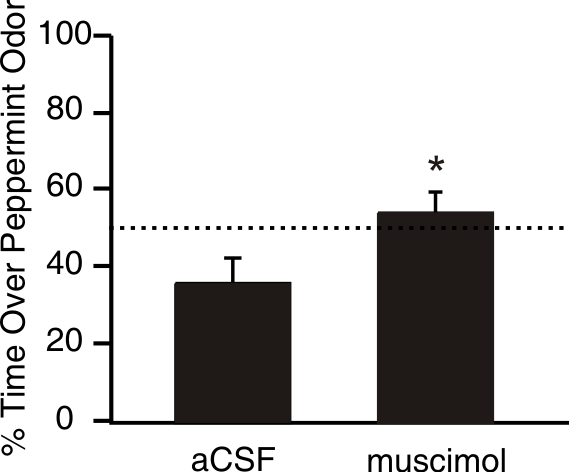

Supplement: Figure S2 — Muscimol lateral bulbar infusion interfered with peppermint odor perception in rat pups. Muscimol (10 mM, 1 µl; N = 6) or vehicle aCSF (N = 6) was infused laterally in the olfactory bulbs. The pups were tested for odor preference 10 min after the infusions. Muscimol-infused pups lost the natural aversive response to peppermint bedding which was shown by the control pups (t = 2.227, p = 0.025). Bars show the percentages of time spent on the peppermint side in a two-choice test box in the two experimental groups. *p<0.05. Error bars, mean±SEM. (TIF) [file pone.0035024.s002.tif]
